# Alternative pre-mRNA splicing as a mechanism for terminating Toll-like Receptor signaling

**DOI:** 10.3389/fimmu.2022.1023567

**Published:** 2022-12-01

**Authors:** Frank Fang Yao Lee, Scott Alper

**Affiliations:** ^1^ Department of Immunology and Genomic Medicine and Center for Genes, Environment, and Health, National Jewish Health, Denver, CO, United States; ^2^ Department of Immunology and Microbiology, University of Colorado School of Medicine, Anschutz, CO, United States

**Keywords:** TLR signaling, innate immunity, inflammation, pre-mRNA splicing, spliceosome, RNA binding protein

## Abstract

While inflammation induced by Toll-like receptor (TLR) signaling is required to combat infection, persistent inflammation can damage host tissues and contribute to a myriad of acute and chronic inflammatory disorders. Thus, it is essential not only that TLR signaling be activated in the presence of pathogens but that TLR signaling is ultimately terminated. One mechanism that limits persistent TLR signaling is alternative pre-mRNA splicing. In addition to encoding the canonical mRNAs that produce proteins that promote inflammation, many genes in the TLR signaling pathway also encode alternative mRNAs that produce proteins that are dominant negative inhibitors of signaling. Many of these negative regulators are induced by immune challenge, so production of these alternative isoforms represents a negative feedback loop that limits persistent inflammation. While these alternative splicing events have been investigated on a gene by gene basis, there has been limited systemic analysis of this mechanism that terminates TLR signaling. Here we review what is known about the production of negatively acting alternative isoforms in the TLR signaling pathway including how these inhibitors function, how they are produced, and what role they may play in inflammatory disease.

## Introduction

### The Jekyll and Hyde nature of TLR signaling and inflammation

The inflammatory response is critical for fighting infection. However, inflammation that is overly robust in an acute setting or inflammation that becomes persistent in a chronic setting can damage tissues and cause disease. Thus, it is not only critical that inflammation be turned on when needed upon infection, but it is also vital that the inflammatory response ultimately is turned off. Numerous epidemiological studies demonstrate the opposing roles of inflammation on infectious and inflammatory disease. For example, patients with rheumatoid arthritis who received anti-TNF therapy for >12 weeks were at a 2-fold increased risk of serious infection (1). Similarly, a large meta-analysis of patients receiving glucocorticoids for > 15 days in the United Kingdom found that these individuals were at a 2-fold increased risk of bacterial skin infection and more than 5-fold increased risk of lower respiratory tract infection (2). These effects are not unexpected and suggest that modulating the maintenance of inflammation rather than ablating the inflammatory response entirely might be a useful therapeutic avenue to explore to treat chronic inflammatory disease without ablating the necessary initial anti-pathogen response.

**Figure 1:**
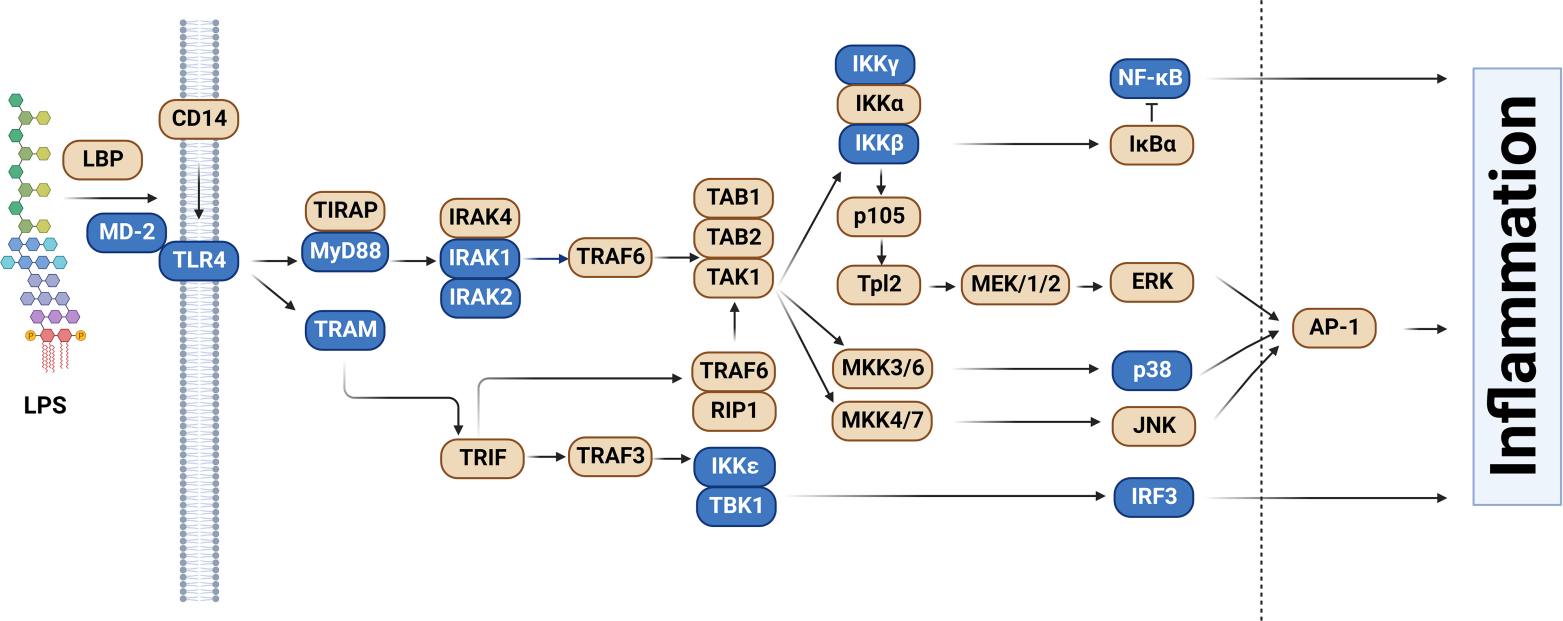
Splice forms that encode inhibitors of signaling in the Toll-like receptor signaling pathway. The schematic depicts the Toll-like receptor (TLR) signaling pathway, adapted and modified from the KEGG pathway database (6, 7). Genes color-coded in blue, in addition to producing the canonical splice forms that encode positive mediators of signaling, also produce alternative mRNA splice forms that encode inhibitors of TLR signaling. Figure created with BioRender.com.

One of the best-understood signaling pathways that induces inflammation is the Toll-like Receptor (TLR) signaling pathway. TLRs are a family of single-pass transmembrane receptors that recognize components of pathogens known as Pathogen-Associated Molecular Patterns (PAMPs) (3, 4). For example, TLR4 and the MD-2 co-receptor recognize lipopolysaccharide (LPS), a component of the coat in Gram negative bacteria (4, 5). Other TLRs recognize other pathogenic components, such as TLR3, which recognizes dsRNA from RNA viruses, and TLR5, which recognizes bacterial flagellin. These TLRs, once activated, initiate downstream signaling events that culminate in the activation of pro-inflammatory transcription factors including NF-κB and AP1 as well as the activation of the interferon response. In the case of LPS, which is one of the best studied PAMPs, binding to LPS leads to dimerization of the TLR4 receptor. This in turn leads to the formation of a large signaling complex that includes but is not limited to the MyD88 signaling adaptor (**Figure 1**), members of a family of IRAK kinases including IRAK4 and IRAK1 and/or IRAK2, and many downstream components. This signaling continues, leading to activation of the pro-inflammatory transcription factors NF-κB and AP1 and inflammatory cytokine production (**Figure 1**). TLR4 also signals through a second signaling adaptor, TRIF, which not only activates NF-κB, but also activates IRF3 and subsequent IFNβ production (**Figure 1**). With the exception of IκBα, which inhibits NF-κB activation, all the depicted components in the canonical TLR signaling pathway are positive effectors of signaling (**Figure 1**).

TLR signaling is required to fight infection as evidenced by numerous mouse (4, 5, 8, 9) and human (10–13) studies. However, persistent TLR signaling can contribute to the pathogenesis of many diseases with an inflammatory component including atherosclerosis, asthma, and cancer (14–17). Thus TLR signaling must be tightly regulated, active when needed and inactive when not. The mechanisms that mediate the activation of TLR signaling have been well studied (4, 5). While the mechanisms terminating this response have received less study, many negative regulators of TLR signaling and NF-κB activation have been identified (18–21). These negative regulators are themselves often induced by inflammatory stimuli such as PAMPs (18–21), and thus production of these negative regulators can be thought of as a negative feedback loop that helps keep inflammation in check in healthy individuals. One representative example of such a negative feedback loop is production of the A20 protein, a de-ubiquitinase that inhibits multiple proteins in the TLR signaling pathway (22–25). A20 production is induced by multiple inflammatory stimuli including LPS, IL-1, and TNF (26–28). Knockout of *Tnfaip3*, the gene that encodes A20, in mice leads to an increased inflammatory response, hyper-responsiveness to LPS stimulation, and premature death (29). Knockout of *Tnfaip3* in specific cells in mice leads to a variety of auto-inflammatory diseases analogous to Systemic lupus erythematosus, inflammatory bowel disease, or rheumatoid arthritis (25). Loss-of-function mutations in *TNFAIP3* in humans cause an early onset auto-inflammatory disease (22–24). These examples illustrate the importance of these negative regulators of TLR signaling in preventing inflammatory disease.

In the current review, we explore the role that alternative pre-mRNA splicing plays in the termination of TLR signaling and inflammation. Intron removal from pre-mRNA is mediated by the spliceosome, a large ribonucleoprotein complex (30–32). The spliceosome is composed of five small nuclear RNAs (snRNAs) called U1, U2, U4, U5, and U6 and associated proteins that interact with each snRNA (generating 5 snRNPs or small nuclear ribonucleoproteins). These snRNPs assemble on key regulatory sites in the pre-mRNA, with U1 binding to the 5’ end of introns and U2 binding to the 3’ end of introns early during the assembly process. Ultimately, the fully mature spliceosome complex mediates intron removal in two trans-esterification reactions. The intron is released in a lariat structure and the flanking exons are ligated together.

**Figure 2:**
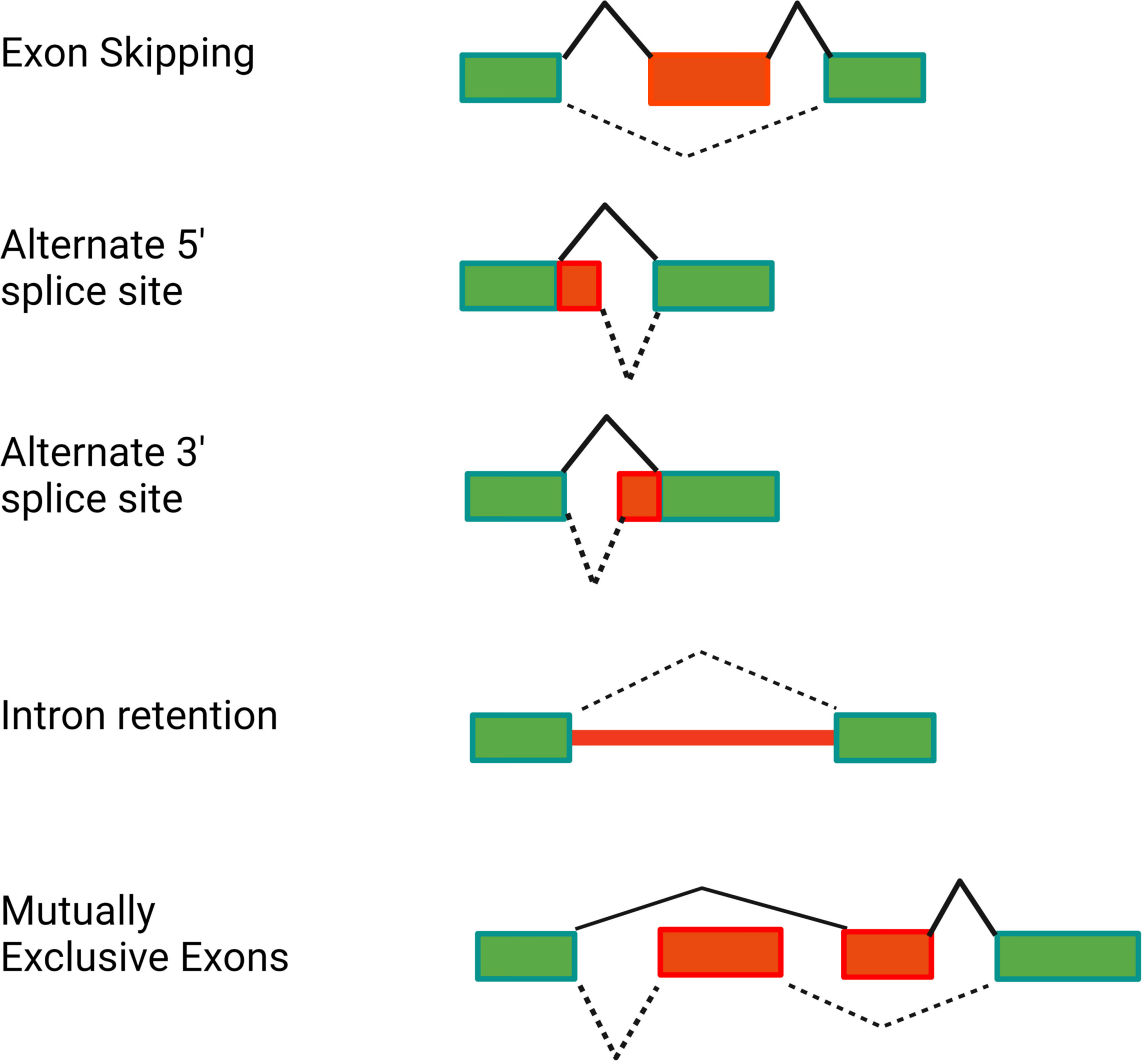
Alternative pre-mRNA splicing can generate several classes of alternative splice forms. The schematics depict different classes of alternative splicing events. Rectangles depict the exons present in the pre-mRNA. Red is used to highlight the relevant regions of the pre-mRNA that are altered in the alternative isoform. Solid black lines indicate splicing of the canonical mRNA. Dotted black lines indicate splicing of the alternative isoform. Figure created with BioRender.com.

More than 95% of human genes are alternatively spliced (33–35). This alternative splicing can produce proteins of varying or even opposing functions, thereby greatly increasing the complexity of the proteome. Alternative splicing can produce a variety of mRNA isoforms by incorporating different exon and intron sequences into the mature mRNA (**Figure 2**). These include exon skipping (in which one exon from the pre-mRNA is skipped and not included in the mature mRNA), intron retention (in which an intron in the pre-mRNA is not removed and is still present in the mature mRNA), alternate 5’ splice site or alternate 3’ splice site usage (in which an intron is removed and exons are ligated together using an alternate splice site), or mutually exclusive exon usage (in which two alternate exons are present in the pre-mRNA but only one of the two alternate exons is retained in the mature mRNA) (**Figure 2**).

The effect of alternative splicing in the immune system is not unique to TLR signaling; for example, alternative splicing is known to affect many genes in the T-cell receptor signaling pathway (36–38). Here we focus on alternative splicing in the TLR signaling pathway. Specifically, we describe how almost all classes of alternate splicing events (**Figure 2**) are used to produce negatively acting splice forms in the TLR signaling pathway.

**Figure 3:**
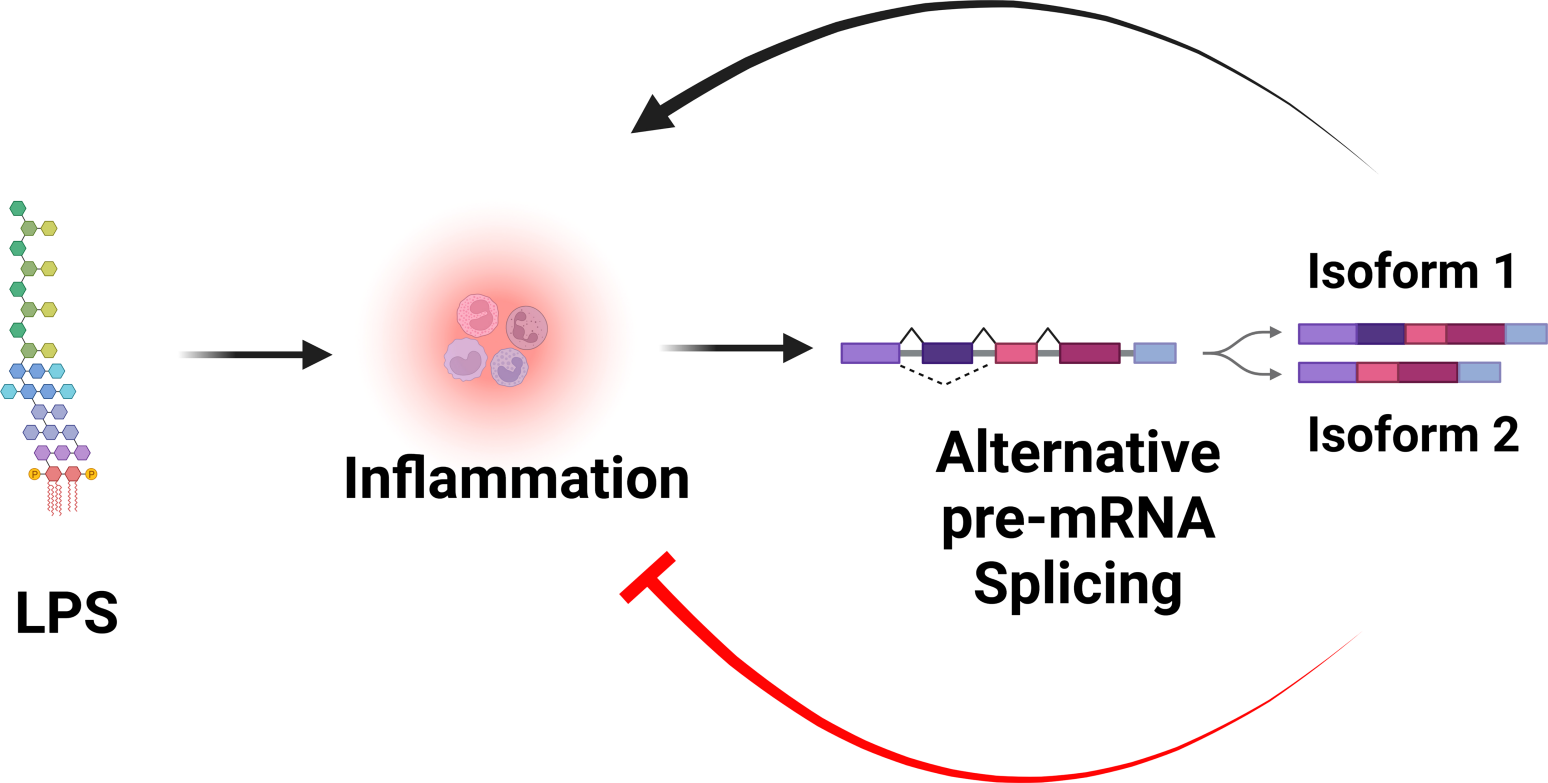
Alternative pre-mRNA splicing mediates a negative feedback loop that limits inflammation. LPS stimulation is sensed by the TLR4 signaling pathway, which induces inflammation. LPS stimulation also alters pre-mRNA splicing of many genes in the TLR4 signaling pathway, leading to the production of splice forms that encode negative regulators that inhibit inflammation. Figure created with BioRender.com.

In addition to producing the canonical positive effectors of TLR signaling, many genes in the TLR signaling pathway also encode alternative splice forms that produce negatively acting proteins that terminate TLR signaling. Moreover, many of these negatively acting splice forms are themselves induced by prolonged inflammatory stimulation. Thus, these splice forms mediate a negative feedback loop that limits inflammation and perhaps prevents disease (**Figure 3**). While there have been many published reports identifying these negatively acting splice forms on a case-by-case basis, there has been little pathway-level analysis of this alternative splicing-mediated negative feedback loop. We speculate that this pathway-level alternative splicing mechanism plays a key role in terminating inflammation and preventing inflammatory disease. In this review, we discuss in detail how alternative pre-mRNA splicing turns TLR signaling off at both the gene and pathway level, what effect this may have on inflammatory disease, what little is known about the mechanisms that mediate this alternative splicing, and discuss the myriad of open questions about this topic.

## Negatively acting splice forms in the TLR signaling pathway

RNAseq studies and individual gene by gene studies in the TLR signaling pathway have demonstrated that PAMP stimulation or infection induces extensive alternative splicing in this pathway (39–48). While these numerous alternative splice forms are reported to serve a myriad of different functions, here we focus solely on those splice forms that convert these canonical positive regulators of inflammation into dominant negative inhibitors of inflammation. We have cataloged 13 genes in the TLR signaling pathway that, in addition to encoding mRNAs that produce the positively acting canonical proteins in the pathway, also produce alternative isoforms that encode negative regulators of signaling (**Figure 1** and **Table 1**). Many but not all of these negative regulators are reported to be induced by PAMP, suggesting that these alternate splice forms may constitute a negative feedback loop(s) that terminates inflammation (**Figure 3**).

**Table 1:** Alternate splice forms that encode negative regulators in the TLR signaling pathway.

Gene	Pro	Anti	Species	Event	Effect of PAMP	Notes	Reference
MD-2	MD-2	MD-2B	Mouse & Rat	54 bp (18 aa) deletion 5’ end exon 3	Not induced by LPS (data not shown)	Inhibits NF-κB reporter, binds TLR4, prevents TLR4 from reaching surface	(49, 50)
MD-2	MD-2	MD-2s	Human	exon 2 skip	Induced by LPS, IFNγ, IL-6	Inhibits LPS-induced NF-κB reporter & IL-8 production, binds LPS & TLR4, competitively inhibits binding of MD-2 to TLR4	(51)
TLR4	TLR4	smTLR4	Mouse	additional exon between exons 2 & 3	Induced by LPS	In frame stop produces truncated soluble protein. Inhibits LPS-induced NF-κB reporter & TNF production	(52)
TLR3	TLR3	unnamed	Human	Inhibitory isoform lacks 1,622 bp	Induced by Type I Interferons	Inhibits poly(I:C)-induced signaling	(53)
MyD88	MyD88-L	MyD88-S	Mouse & Human	exon 2 skip	Induced by multiple stimuli – see **Figure 5**	Inhibits LPS and IL1β-induced NF-κB activation.	(54–58)
TRAM	TRAM	TAG	Human & other primates	alternate splice site in exon 4	Decreased 1 hr post-LPS, then rebounds by 2 hrs in HEK293 cells expressing TLR4, MD2, and CD14	Inhibits LPS-induced ISRE but not NF-κB reporter. Inhibits TRAM by displacing TRIF	(59)
IRAK1	IRAK1	IRAK1c	Human	exon 11 skip	Induced by LPS in monos & DCs	Lacks kinase activity, not phosphorylated by IRAK4, can bind IRAK2, MyD88, Tollip, and TRAF6. Inhibits NF-κB, AP1, and LPS/CpG-induced cytokine production.	(60)
IRAK2	IRAK2	IRAK2c	Mouse	Alternate transcription start site leads to loss of exons 1-3, longer 5’UTR in exon 4	Induced by LPS	Inhibits NF-κB	(61)
IRAK2	IRAK2	IRAK2d	Mouse	exon 2 skip & 30bp deletion 5’ end of exon 12	Not reported	Inhibits NF-κB	(61)
IKKγ	IKKγ	IKKγΔ	Human	exon 5 skip	Induced by virus infection but not as much as WT isoform	Complex. May depend on cell type - see text.	(62–64)
IKKβ	IKKβ	IKKβb	Mouse & Human	Intron 15 retention	Not reported	siRNA indicates is negative regulator.	(65)
TBK1	TBK1	TBK1s	Mouse & Human	exon 3-6 skip	Induced by Sendai virus (SeV) infection	binds RIG-I, disrupts RIG-I-VISA interaction, inhibits IRF3 activation	(66)
IKKε	IKKε	IKKε-sv1	Human	exon 21 skip	Induced by TNF	Forms dimers with IKKε-wt, inhibits IKKε-induced IRF3 signaling including anti-viral activity. Fails to interact with TANK, NAP1, or SINTBAD.	(67)
IKKε	IKKε	IKKε-sv2	Human	exon 20 skip	Induced by TNF	Forms dimers with IKKε-wt, inhibits IKKε-induced IRF3 signaling including anti-viral activity. Fails to interact with TANK, NAP1, or SINTBAD.	(67)
NF-κB p65	p65	p65Δ	Mouse & Human	Alternate splicing deletes 30 bp (10 aa) at 5’ end of an exon	Not reported	Inhibits NF-κB dimerization and NF-κB activity	(68, 69)
p38α	p38α	EXIP	Mouse & Human	exon 10 and 11 skip	Not reported	Inhibits NF-κB activation	(70)
IRF3	IRF3	IRF3a	Human	Additional exon between exons 2 & 3	Not reported	Inhibits virus-induced IRF3 activity and IFNβ expression	(71, 72)
IRF3	IRF3	IRF3-CL	Human	Alternate 3’ splice site leads to the addtion of 16 bp to 5’ end of exon 7	Not reported	Inhibits virus-induced IRF3 activity and IFNβ expression	(73)
IRF3	IRF3	IRF3-nirs3	Human	exon 6 skip	Not reported	Inhibits virus-induced IRF3 activity and IFNβ expression	(74, 75)
IRF3	IRF3	IRF3f	Human	exon 2 skip	Not reported	Inhibits IRF3	(74)

“Pro” is name of the canonical pro-inflammatory isoform. “Anti” is the name of the alternative splice form that encodes the negative regulator of signaling.

## Alternative pre-mRNA splicing of MyD88 produces a dominant negative inhibitor of NF-κB

By far the most studied negatively acting isoform in the TLR signaling pathway is an isoform produced by the *MyD88* gene. MyD88 is a signaling adaptor that mediates the response to all TLRs except for TLR3. MyD88 also mediates the response downstream of the IL1β receptor; the IL-1 receptor and Toll-like receptors utilize many common signaling components. The *MyD88* gene in mice encodes a 5 exon mRNA that produces the canonical positive regulator of inflammation. Exon 1 encodes the death domain (DD) that interacts with the DD in the downstream IRAK1 kinase. Exon 2 encodes an intermediate domain (ID), and exons 3-5 encode the Toll-Interleukin 1 Receptor (TIR) domain that interacts with Toll-like receptors and the IL-1 receptor. Dimerization of TLRs leads to recruitment and multimerization of MyD88, recruitment of the IRAKs, and thus formation of a large signaling complex known as the Myddosome (76–78).

In addition to the canonical long MyD88 isoform (MyD88-L), alternative splicing of the MyD88 pre-mRNA also produces a shorter isoform (MyD88-S) in which exon 2 is skipped. This short isoform contains an in frame 135 bp deletion that still produces a functional protein. This short isoform has been observed in mice, humans, and other mammals, and in multiple tissues and cell types including monocytes, macrophages, dentritic cells, T cells, B cells, epithelial cells, and glial cells. Thus, like the canonical TLR signaling pathway components, MyD88-S is likely present in most tissues and cell types in the body.

**Figure 4:**
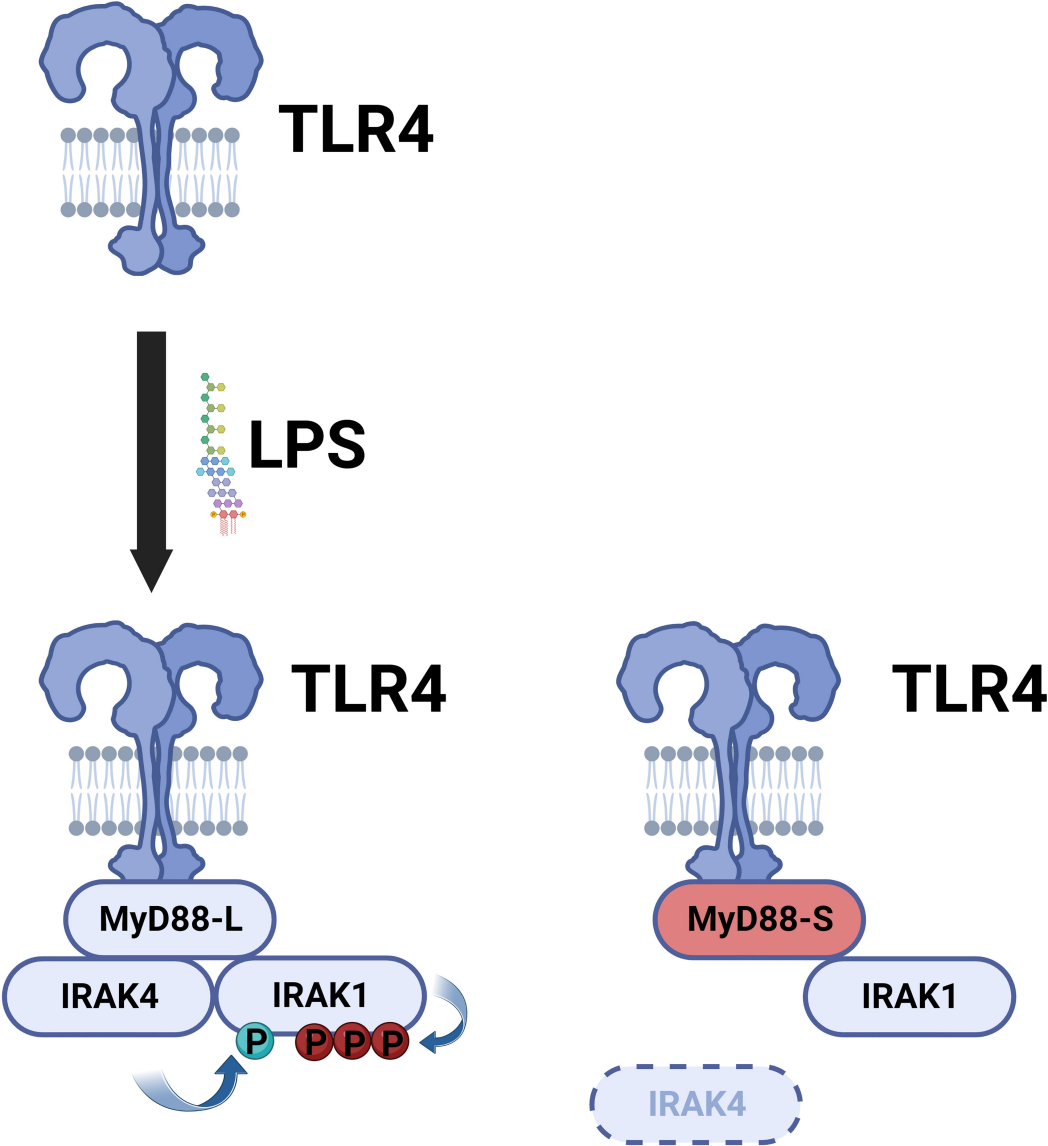
MyD88-S inhibits TLR signaling by preventing phosphorylation of IRAK1. Upon LPS stimulation, TLR4 recruits MyD88-L to the complex. MyD88-L in turn recruits IRAK4 and IRAK1. IRAK4 phosphorylates IRAK1, and IRAK1 auto-phosphorylates itself. MyD88-S is able to interact with TLR4 and IRAK1 but not IRAK4. Thus, MyD88-S prevents IRAK1 phosphorylation. For simplicity, not every protein in the signaling complex is depicted. Moreover, this signaling complex contains multiple copies of each peptide; for simplicity, only a single copy of each peptide is depicted in the diagram. Figure created with BioRender.com.

MyD88-L serves as a signaling adaptor that bridges signaling between the TLRs and the downstream IRAK kinases, ultimately leading to the activation of the NF-κB and AP1 transcription factors. In contrast, MyD88-S serves a very different function. MyD88-S, which was first described around 20 years ago, is a dominant negative inhibitor that prevents NF-κB activation (54). In that first study, the authors demonstrated that overexpression of MyD88-S inhibits IL-1 but not TNF-induced NF-κB activation in HEK293T cells. The IL-1R signaling pathway, but not the TNFR pathway, shares many similar signaling components to the TLR signaling pathway, including the MyD88 signaling adaptor, and thus many studies likely relevant to TLR signaling were first performed with IL1 stimulation. MyD88-S overexpression also partially inhibited LPS-induced NF-κB activation in MF4/4 macrophages (54). MyD88-S was able to bind to the IL-1R and MyD88-L, and moreover, MyD88-S competed with MyD88-L for binding to the IL-1R. MyD88-S, like MyD88-L also bound to the IRAK1 kinase, but unlike MyD88-L, which promotes IRAK1 phosphorylation, MyD88-S inhibited IRAK1 phosphorylation (54). The reason for this key difference is that MyD88-L but not MyD88-S binds the IRAK4 kinase (55). This inability of MyD88-S to bind to IRAK4 is because of the absence of key IRAK4 interacting residues in MyD88-S, which stalls growth of the Myddosome and IRAK1 activation (**Figure 4**) (79, 80). This inability of MyD88-S to activate IRAK1 likely explains its dominant-negative effect.

Intriguingly, while MyD88-S inhibits IL-1 and TLR-induced NF-κB activation, it does not inhibit AP1 activation (81). In fact, overexpression of MyD88-S can actually increase phosphorylation of JNK and resulting AP1 activity in HEK293T cells (81). How MyD88-S is able to exert differential effects on NF-κB and AP1 and what the consequences of these differential effects are remains to be determined.

These various studies investigated the effect of MyD88-S overexpression. Vickers et al. (82) used a different approach to modulate MyD88 splicing. They developed splice-switching antisense oligonucleotides (AONs) that target the exon-intron boundaries in MyD88, and that as a consequence, induced exon 2 skipping. In other word, these AONs drive up the expression of MyD88-S at the expense of MyD88-L. These AONs likewise demonstrate that MyD88-S is a pathway inhibitor, weakening the response to Il-1β and to CpG (A TLR9 agonist) treatment (82). Also consistent with the demonstration that overexpression of MyD88-S weakens TLR signaling are studies that inhibited MyD88-S using an isoform specific siRNA that bracketed the unique exon 1-exon 3 junction in MyD88-S. MyD88-S inhibition in mouse RAW264.7 macrophages led to an increase in LPS-induced inflammatory cytokine production (83).

The inhibitory effect of MyD88-S is not unique to immune cells. MyD88-S also acts as a negative regulator in epithelial cells (56, 84) and glial cells (85). For example, in BEAS-2B human epithelial cells, MyD88-S overexpression inhibits Non-typeable Haemophilus influenzae (NTHi)-induced NF-κB activation and cytokine production; in contrast, MyD88-S-specific siRNA treatment led to increased NF-κB activation and inflammatory cytokine production in these epithelial cells (56, 84).

**Figure 5:**
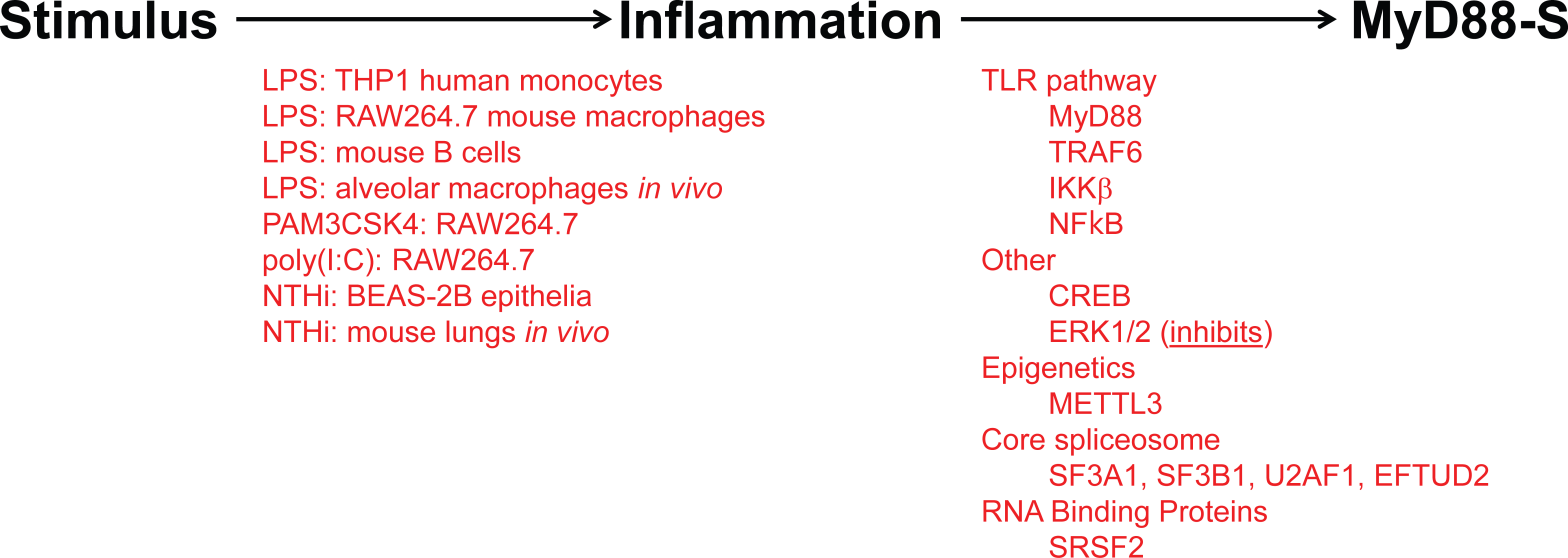
Inflammatory stimuli and signaling proteins that regulate MyD88-S production. On the left (in red) are listed all the inflammatory stimuli that have been reported to induce MyD88-S expression. On the right (in red) are listed all the proteins that have been reported to mediate the stimuli-induced increase in MyD88-S expression.

As observed for many negative regulators in the TLR signaling pathway, prolonged PAMP stimulation leads to increased production of MyD88-S (**Figure 5**). This was first reported for THP1 human monocytes, in which LPS increased MyD88-S levels (54). Multiple PAMPs have since been shown to increase MyD88-S levels, including LPS (TLR4 agonist), PAM3CSK4 (TLR2 agonist) and poly(I:C) (TLR3 agonist), which all increase MyD88-S levels in RAW264.7 macrophages (57). It is interesting to note that the TLR3 agonist increases MyD88-S levels, as TLR3 is the only Toll-like receptor to not use MyD88 as a signaling adaptor. This suggests that there may be some cross-talk between MyD88-independent and MyD88-dependent signaling pathways.

LPS stimulation also increases MyD88-S production in isolated mouse B cells (58). Finally, LPS also alters MyD88 splicing *in vivo*, as intratracheal instillation of LPS increases MyD88-S production in alveolar macrophages in mice (57). There is even some evidence that LPS instillation in lungs in human volunteers can increase MyD88-S levels, although this increase did not reach statistical significance (86), perhaps because of the small study size. Infection has also been shown to increase MyD88-S levels, as Non-typeable Haemophilus influenzae (NTHi) infection has been shown to increase MyD88-S in lung epithelial cells in culture and in whole mouse lungs *in vivo* (56).

In these various studies, while there was a profound increase in MyD88-S levels following PAMP stimulation, there was typically not much of a compensatory decrease in MyD88-L levels. This likely reflects the relative levels of MyD88-L and MyD88-S. In an unstimulated cells, there is far more MyD88-L than MyD88-S; however, this small quantity of MyD88-S is still capable of inhibiting signaling non-stoicheometrically (54, 83), possibly by poisoning production of the whole Myddosome (80).

## Other splice forms in the TLR signaling pathway that produce negative regulators

In contrast to MyD88-S, which has been studied in some detail, there is far less information available about the other negatively acting splice forms in the TLR signaling pathway (**Table 1**). Thus, there is less information about the mechanisms by which these splice forms act, whether or not PAMP stimulation changes the levels of these isoforms, and sometimes unclear information about the conservation of these different isoforms in different mammalian species.

TLR4 and MD-2, the two co-receptors for LPS, are a good case in point (**Table 1**). Alternate splice forms of MD-2 that inhibit signaling have been identified in both mice and humans, but these alternate splice forms are different in these two species. The alternate isoform MD-2B found in mice and rats is formed by a 54 base pair deletion at the 5’ end of exon 3. MD-2B inhibits LPS-induced NF-κB activation (49, 50). MD-2B still binds to TLR4 but prevents TLR4 from reaching the cell surface, offering a potential mechanism for how MD-2B inhibits signaling (50). MD-2B is reported to not be induced by LPS, but this was presented as data not shown (50). In some cases, LPS induction of these negatively acting isoforms can require very prolonged stimulation times, so timing is relevant, and it is unclear what time points the investigators assessed.

The negatively acting form of MD-2 in humans has been named MD-2 short or MD-2s (51). MD-2s differs from mouse MD-2B, and instead is produced by an exon 2 skipping event. This alternate isoform results in the deletion of 30 amino acids and the change of one junction-encoded amino acid (D38G); the authors were unable to identify this isoform in mice (51). MD-2s inhibits LPS-induced NF-κB activation and resulting IL-8 production. The mechanism of inhibition used by MD-2s also differs from that of MD-2B. MD-2s can bind to TLR4 and can compete with wild type MD-2 for binding to TLR4 (51). Moreover, unlike MD-2B, MD-2s levels are reported to be induced by LPS (as well as IFNγ and IL-6) (51). Thus, negatively acting splice forms of MD-2 are present in both mice and humans, but there are some differences in the precise splicing change, the mechanism of action of the negative regulator, and the control of production of the negative regulator between the two species.

The negatively acting form of TLR4, the other component of the LPS receptor, also illustrates some of the unknowns in terms of species conservation. The negatively acting form of TLR4 identified in mice is called smTLR4 for soluble mouse TLR4. The mouse *Tlr4* gene produces a 3 exon mature mRNA; smTLR4 is encoded by an alternative splice form that involves the inclusion of an additional alternative exon between exons 2 and 3 (52). This introduces an in frame stop codon, resulting in a truncated TLR4 protein of only 86 amino acids common to TLR4 plus 36 novel amino acids (52). smTLR4 mRNA encodes a soluble protein that inhibits LPS-induced NF-κB activation and TNF production. LPS stimulation induces smTLR4 production in mouse RAW264.7 macrophages, so smTLR4, like MyD88-S and MD-2s, may be part of a negative feedback loop that limits inflammation. smTLR4 was also induced in T cells following anti-CD3 treatment, indicating that other modes of stimulation in other cell types also induce production of this negative regulator (52). It remains speculative how smTLR4 inhibits the LPS response.

So is there an analogous negatively acting splice form of TLR4 in humans? The answer is somewhat murky. There is no direct homolog of this alternate mouse exon present in the human *TLR4* gene (52). However, there are human TLR4 transcript variants that are predicted to produce a similar truncated protein that could exert a similar negative effect (52, 87), although this hasn’t been tested experimentally. At the protein level, soluble forms of TLR4 (as well as other TLRs) have been detected in various human tissues (88–95). These soluble TLR4 proteins act as negative regulators of signaling (96, 97). Proteolytic processing of TLR4 is known to be involved in production of sTLR4 (98–102); it is unknown if altered splicing of TLR4 also contributes to sTLR4 production in humans.

The IRAK1 and IRAK2 kinases that function downstream of MyD88 also illustrate differences in production of negatively acting splice forms in different mammals, with the IRAK1 negatively acting form specific to humans and the negatively acting forms of IRAK2 specific to mice. IRAK1 and IRAK2 are partially redundant kinases that are recruited to the Myddosome after LPS stimulation, with IRAK1 acting immediately after LPS stimulation and IRAK2 functioning at later times after LPS challenge (103, 104). The *IRAK1* gene encodes multiple mRNAs, but only one of these different isoforms, IRAK1c, encodes a negative regulator (60, 105, 106). IRAK1c, which is present in humans but not mouse cells, is generated by an exon 11 skipping event. IRAK1c protein can still bind to MyD88, but IRAK1c is not phosphorylated by IRAK4, and IRAK1c lacks kinase activity of its own (60). Thus, IRAK1c is a dominant negative inhibitor that prevents IL-1β-induced NF-κB and AP1 activation and that prevents IL-1β, LPS, and CpG-induced production of pro-inflammatory cytokines (60). The precise mechanism by which IRAK1c exerts this inhibitory effect remains unclear. LPS stimulation leads to increased production of IRAK1c in both monocytes and dendritic cells (60). Both isoforms of IRAK1 are found in CD4^+^ Th cells, but IRAK1c is the only form of IRAK1 detected in activated CD4^+^ Th cells (107), suggesting that changes in IRAK1 splicing could modulate T cell activity. Interestingly, while IRAK1c is found in young brains, IRAK1 is the predominant isoform found in older brains; this raises the possibility that the anti-inflammatory effects of IRAK1c are lost as the brain ages in older individuals (108).

The mouse *Irak2* gene encodes four different isoforms termed Irak2a, Irak2b, Irak2c, and Irak2d (61). These alternate isoforms are not found in humans cells. Of these, Irak2c and Irak2d inhibit NF-κB activity (61). Irak2d is generated by two splicing changes, the skipping of exon 2 and the use of an alternate splice acceptor site that lies 30 bp into exon 12. Irak2c is produced by the use of an alternate transcription start site; thus Irak2c lacks exons 1-3 and contains an extended 5’UTR in exon 4. So while Irak2c and Irak2d are both inhibitory isoforms of Irak2, formally only Irak2d is produced by alternative splicing of the Irak2 pre-mRNA. Irak2c was induced by LPS treatment; the authors did not assess the effect of LPS on Irak2d levels (61). How these two isoforms inhibit NF-κB activation is unknown, although both isoforms lack the death domain, so it is possible that the interaction with Irak4 is perturbed in some fashion.

Extending downstream in the singling pathway, multiple alternative splicing events produce negative regulators that control NF-κB activity. In unstimulated cells, NF-κB is held in an inactive complex in the cytoplasm by IκBα. Upon stimulation, the IKK complex phosphorylates IκBα, leading to the ultimate proteolysis of IκBα; this releases NF-κB, which translocates into the nucleus and activates gene expression (109). The IKK complex is composed of three subunits, IKKα and IKKβ, which are catalytic subunits, and IKKγ, also known as Nemo, a regulatory subunit.

A negatively acting isoform of IKKβ has been identified. This isoform, named IKKβb, retains intron 15 in the mature mRNA (65). This change introduces a premature stop codon that truncates the COOH-terminus of the protein. As outlined below, the *IKKε* gene produces negatively acting isoforms that likewise produce a truncated protein, suggesting that IKKβb may likewise act as an inhibitor. Indeed, siRNA-mediated inhibition of IKKβb led to increased LPS-induced cytokine production (65). An analogous isoform of IKKβb is present in humans, although this isoform has not been studied. It is unknown mechanistically how the IKKβb isoform acts or if LPS stimulation regulates its production, so many questions about this isoform remain.

An alternate isoform of Nemo/IKKγ has also been identified in which exon 5 is skipped; this isoform, known as IKKγΔ, is an in-frame 153 bp deletion. The effect of this isoform is complex, and whether to classify it as a negative regulator or not is somewhat unclear, as different results have been reported depending on the nature of the stimulus, the cell type being studied, and the inflammatory readout monitored. In some contexts, IKKγΔ acts much like the wild type isoform. IKKγΔ can bind to IKKγ and still mediates signaling and activation of NF-κB induced by IKKβ, IKKα, and TNF stimuli (62). In contrast IKKγΔ did not mediate HTLV-1-Tax induced NF-κB activation (62). HTLV-Tax is known to couple to IKKγ to activate NF-κB (110). In fact, IKKγΔ acted as a dominant negative inhibitor of HTLV-1 Tax-induced NF-κB activity (62). A follow-up study further demonstrated the complexity of the function of this isoform. In studies in which either IKKγ or IKKγΔ were reconstituted into IKKγ knockout mouse embryonic fibroblasts (MEFs), the authors found that IKKγΔ successfully mediated virus-induced NF-κB activation but was unable to mediate virus-induced IRF3 activation (63). Thus, wild type IKKγ but not IKKγΔ MEFs were deficient in virus-induced Type I interferon production and the ability to control the viral infection (63). The authors went on to demonstrate that IKKγΔ acted as a dominant negative inhibitor of virus-induced Type I interferon production, perhaps because of the inability of IKKγΔ to interact with the TBK1 adapter protein TANK (63). The IKKγΔ variant isoform is induced by virus infection, but the wild type isoform is also induced to an even higher level (63).

As outlined in Section *Do these negative regulators affect disease?* below, recently, a novel human autoinflammatory disease has been described in which heritable mutations drive exon 5 skipping in IKKγ. Studies with human patient samples (64) likewise demonstrate complicated effects that in some cases differ from these prior studies. Expression of IKKγΔ in dermal fibroblasts correlated with a dampened antiviral response; however, in macrophages and T cells, IKKγΔ led to an increase in NF-κB activity and Type I interferon production (64). Thus, overall, it may be better to treat this unique IKKγ isoform as a change of function variant rather than a true negative regulator, although it can act as a negative regulator of signaling in some contexts.

As we continue to work our way down the TLR-NF-κB signaling pathway, we find that the NF-κB p65 subunit itself is alternatively spliced (68, 69, 111–114) and can produce a negatively acting isoform called p65Δ (68, 69). p65Δ, which is found in mice and humans, uses an alternate splice acceptor site that deletes amino acids 222-231 from the protein (68, 69). p65Δ inhibits binding of NF-κB to its promoter target sites, perhaps because this isoform is unable to dimerize efficiently and may therefore prevent p65 binding to DNA (68, 69).

The MyD88-dependent arm of the TLR signaling pathway, in addition to activating NF-κB, also activates the AP1 transcription factor by activating a family of MAP kinases including p38. The p38α gene produces a negatively acting isoform called EXIP (70, 115). EXIP is formed by an exon 10 and 11 skipping event. Strong overexpression of EXIP inhibits NF-κB activity (although more moderate overexpression did the opposite) (70). The mechanisms explaining how EXIP functions have not been explored.

Thus far, we have largely focused on the MyD88-dependent arm of the TLR4 signaling pathway, which leads to activation of NF-κB and AP1. However, TLR4 signaling can also activate MyD88-independent signaling pathways that in addition to activating NF-κB and AP1, also induces Type 1 interferon production. Alternative pre-mRNA splicing has also been reported to produce negatively acting splice forms that encode negative regulators in this arm of the TLR signaling response (**Figure 1** and **Table 1**). Acting most receptor proximal in this arm is an alternative splice form of the adaptor protein TRAM (59). The canonical TRAM mRNA is composed of four exons that encode a protein of 235 amino acids. This protein activates signaling. An alternative splice form of TRAM produces an isoform known as TAG (TRAM Adaptor with Gold domain) (59). This isoform, where exon 3 is spliced into a downstream site in exon 4, produces a protein of 404 amino acids with a novel Gold domain at the NH_2_-terminus connected to the TRAM TIR domain at the COOH-terminus. The Gold domain, or Golgi dynamics domain, controls the subcellular localization of proteins. In HEK293 cells engineered to express CD14, TLR4, and MD2, the authors observed a transient decrease in TAG levels following LPS treatment and a subsequent rebound in TAG levels (59). So LPS does not induce production of this negatively acting isoform. TAG inhibits the MyD88-independent arm of the TLR4 response; in particular, TAG inhibits LPS-induced activation of IRF3 but not IL-8 (59). Mechanistically, TAG is able to displace TRIF from the TRAM signaling adaptor, thereby inhibiting signaling (59).

Further downstream in the TRIF arm of the TLR signaling pathway, two IKK family members, TBK-1 and IKKε, activate the IRF3 transcription factor. Both these IKK family members produce alternatively spliced isoforms that encode inhibitors of signaling (67) as well as alternate isoforms that are not inhibitory (116). Koop et al. (67) reported the identification of two inhibitory isoforms of IKKε that they termed IKKε-sv1 and IKKε-sv2 (sv=splicing variant). IKKε-sv1 is generated by an exon 21 skipping event which leads to the deletion of 25 amino acids near the COOH-terminus of the protein. IKKε-sv2 is generated by an exon 20 skipping event that results in a frameshift and premature stop codon, which leads to a truncation at the COOH-terminus of the protein after 13 novel amino acids. Both splice variants encode proteins that form dimers with wild type IKKε. Both these IKKε variants inhibit wild type IKKε-induced IRF3 activation, possibly because these variant proteins fail to interact with the adapter proteins TANK, NAP1, and SINTBAD (67). TNF stimulation led to increased expression of both negatively acting isoforms, depending on the cell line tested (67).

A negatively acting isoform of TBK1, termed TBK1s, is found in mice and humans, and is formed by an exon 3-6 skipping event (66). This variant has an alternative translation start site that produces a protein with a deletion in the kinase domain (66). TBK1s can bind to RIG-I and disrupt its interaction with VISA (66). Thus TBK1s overexpression inhibits IRF3 nuclear translocation and inhibits Sendai virus (SeV)-induced IFNβ production (66). While the authors focus on RIG-I signaling, based on the nature of these signaling pathways, it seems likely that TLR signaling could also be affected by TBK1s. SeV infection itself increases TBK1s levels, raising the possibility of a potential negative feedback loop (66). Finally, we note that several other negatively acting isoforms of TBK-1 have been identified in zebrafish (117, 118), suggesting that inhibition of TBK1 signaling by alternative pre-mRNA splicing could be an evolutionarily conserved mechanism.

The IRF3 transcription factor becomes active by dimerizing, translocating into the nucleus, and activating gene expression. Several isoforms of IRF3 have been identified that encode proteins that inhibit signaling (71–75). The first such negatively acting form that was described was called IRF3a. IRF3a is produced by the inclusion in the mature mRNA of an alternate exon between exons 2 and 3. This changes the NH_2_-terminus of the protein and deletes much of the DNA binding domain. IRF3a inhibits virus-induced production of IFNβ, likely because IRF3a can bind to and inhibit wild type IRF3 (71, 72). The IRF3-CL isoform is generated by the use of an alternate 3’ splice site that leads to the addition of 16 bp to the 5’ end of IRF3 exon 7. This isoform encodes a protein with a unique COOH-terminus (73). Like IRF3a, IRF3-CL inhibits virus-induced IFNβ production (73). IRF3-CL did not interact with wild type IRF3 protein in unstimulated cells, but did interact with IRF3 when cells were activated by IKKε overexpression, suggesting a possible mechanism for how this variant might inhibit signaling (73). IRF3-nirs3 (also called IRF3e) was identified in hepatocellular carcinoma; this isoform is generated by an exon 6 skipping event, and also produces a negative regulator that inhibited virus-induced IFNβ production (74, 75). Even less is known about still a fourth potential negatively acting splice form, IRF-3f, that also may inhibit wild type IRF3 function (74).

*TLR4* is not the only TLR gene that can generate an inhibitory isoform. A TLR3 inhibitory isoform whose production is induced by type I interferon has been identified in human astrocytes (53). This isoform, which inhibits IRF3 activation, lacks 1,622 bp of the full 3,057 bp TLR3 mRNA (53). This transcript includes a novel stop codon that produces a truncated protein plus 11 novel amino acids. Overexpression of this variant inhibited poly(I:C)-induced phosphorylation of IRF3, IκBα, and STAT1 and therefore inhibited production of IP-10 (53). Like many other negatively acting isoforms, the negatively acting form of TLR3 is induced by inflammatory stimuli – in this case, Type I interferons (53).

In summary, numerous alternative splice forms have been described that encode proteins that inhibit TLR signaling. These alternate splice forms can be generated by a variety of alternative splicing events, most commonly exon skipping, but other alternative splicing events such as altered 3’ splice site usage, intron retention, and alternative exon inclusion also produce negative regulators. These negative regulators have been reported to inhibit many aspects of signaling, although much remains unknown about the extent of these effects and the underlying mechanisms.

## Mechanisms that regulate production of these negatively acting splice forms

PAMP or infection is reported to increase the production of many of these negative regulators, leading to the hypothesis that production of these negative regulators represents a negative feedback loop(s) that terminates persistent pro-inflammatory signaling. Analogous to the study of how MyD88-S regulates TLR signaling, far more has been learned about the mechanisms controlling production of MyD88-S than production of the other negative regulators that have been identified (**Figure 5**).

LPS stimulation is sensed by TLR4 and the downstream TLR4 signaling pathway, so it is logical that some component(s) in this pathway will mediate the effects of LPS on the induction of MyD88-S levels. Indeed, studies that activated and inhibited different components in the TLR4 signaling pathway demonstrated that the entire signaling pathway including NF-κB itself is necessary and sufficient for LPS-induced MyD88-S production in murine macrophages (57). Consistent with these macrophage results, IKKβ (which activates NF-κB), is also required for NHTi-induced MyD88-S production in epithelial cells (56). Other signaling factors that have been reported to regulate MyD88-S production include CREB, which facilitates MyD88-S production in epithelial cells, and ERK1/2, which inhibit MyD88-S production in epithelia (56). The METTL3 epigenetic regulator had a fairly moderate effect on MyD88-S production in dental pulp cells (119), although it is unclear how direct this effect might be.

Thus, perhaps not too surprisingly, components of the TLR4 signaling pathway and a few other signaling and epigenetic regulators have been identified that regulate MyD88 alternative splicing. Ultimately, these signaling regulators must impact the splicing machinery to modulate MyD88 splicing. As described in the Introduction, removal of introns from pre-mRNA is mediated by the spliceosome, which is composed of five small nuclear RNAs (snRNAs) called U1, U2, U4, U5, and U6 and associated proteins that interact with each snRNA (generating 5 snRNPs or small nuclear ribonucleoproteins). Alternative splicing, in turn, can be regulated by RNA binding proteins that modulate spliceosome activity. For example, members of the SRSF (Serine and Arginine Rich Splice Factor) bind mRNAs, and often favor inclusion of the bound exon in the mature mRNA; in contrast, members of the HNRNP (Heterogeneous Nuclear Ribonucleoprotein) often, but not always, will bind to pre-mRNA and will inhibit exon inclusion (120–124).

The core spliceosome regulates MyD88 alternative splicing. siRNA-mediated inhibition of core spliceosome components associated with the U2 snRNP (including SF3A1, SF3B1, and U2AF1) or U5 snRNP (Eftud2) in mouse and/or human macrophages weakens LPS-induced inflammatory cytokine production, in part, by upregulating production of MyD88-S (83, 125, 126). Pharmacological inhibition of SF3B1 likewise increased MyD88-S levels and weakened LPS-induced inflammatory cytokine production, further validating the importance of the core spliceosome in regulating MyD88-S production (83).

Interestingly, inhibition of core splicing components also upregulates other negatively acting splice forms including smTLR4 and IKKβb, and these negatively acting splice forms also regulate LPS-induced cytokine production when the spliceosome is inhibited (65). Our interpretation of these results is that, because of their functional significance, TLR pathway genes have evolved “weak” splicing regulatory sequences that allow for easy splice switching to enable efficient terminate of inflammation. Several studies are consistent with this hypothesis. First, as outlined above, inhibition of the core spliceosome (at ~ the 80% level, monitoring mRNA or protein) weakens LPS-induced inflammatory cytokine production without altering macrophage viability or the ability to phagocytose bacteria (83, 125, 126). However, knockout of these core spliceosome components in mice is lethal (127, 128). We speculate that these results indicate that TLR signaling pathways are more sensitive to spliceosome inhibition than are other essential signaling pathways in immune cells. Consistent with this differential sensitivity are dose response studies using the SF3B1 inhibitory compound spliceostatin A. High doses of this inhibitor are, as expected, lethal; however, low doses that do not affect cell viability or macrophage phagocytic ability still weaken LPS-induced inflammatory cytokine production (83). Pathway analyses of an RNAseq study in which SF3a1 was inhibited in macrophages likewise demonstrates that innate immune signaling pathways are particularly sensitive to spliceosome inhibition (65). All these data are consistent with the idea that the sequences in these TLR pathway genes that control alternative splicing are relatively “weak” allowing for easy splice switching when the proper environmental stimuli are present.

**Figure 6:**
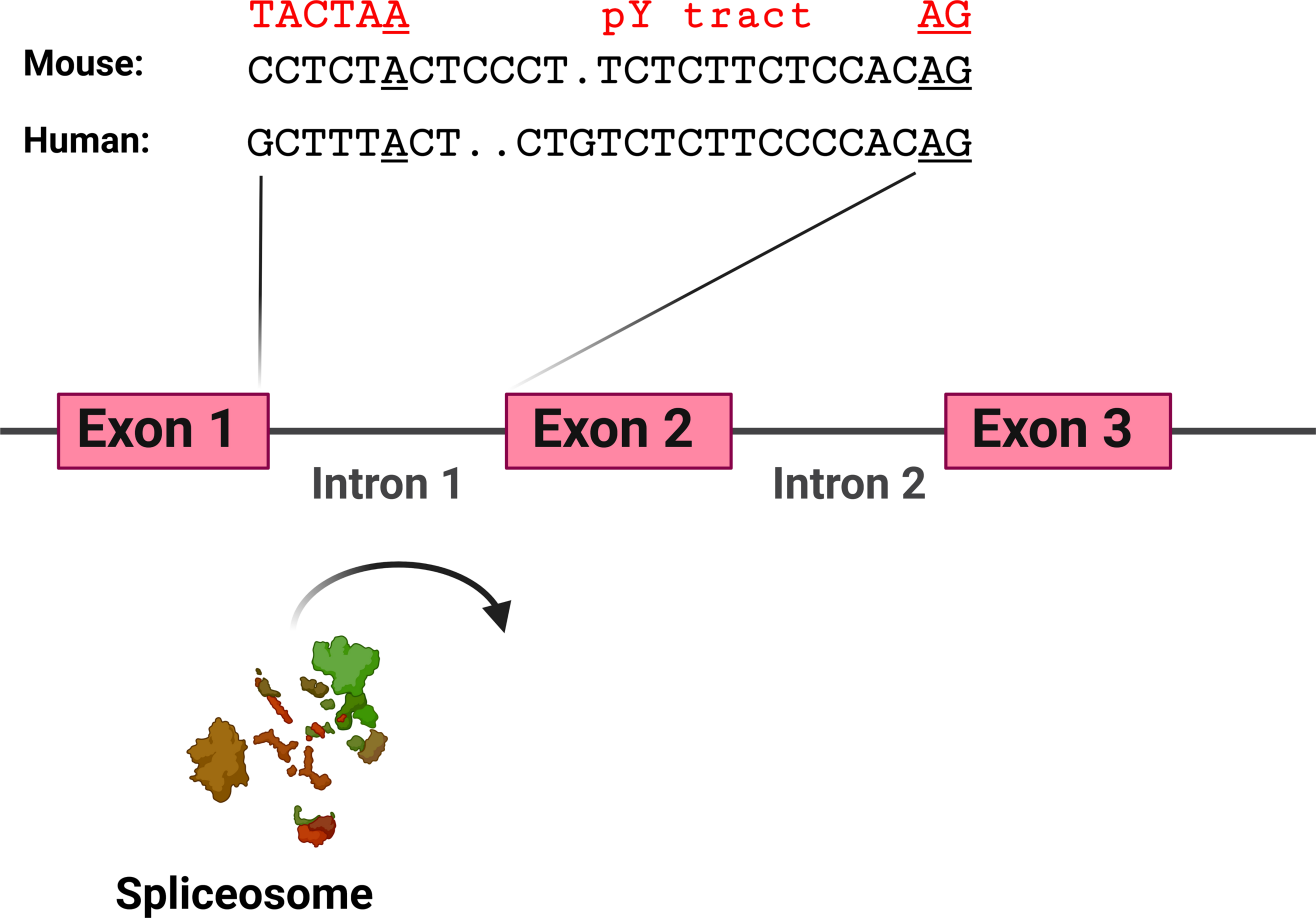
MyD88 intron 1 has relatively weak splicing regulatory sequences. Depicted are the sequences at the 3’ end of intron 1 in mouse and human MyD88. Sequences involved in facilitating exon 2 inclusion include the AG dinucleotide at the 3’ end of the intron, the polypyrimidine (pY) tract upstream of the AG dinucleotide, and the branch point sequence [identified for human MyD88 in (132)] including the branch point adenosine residue underlined. In red above the branch point is a strong branch point sequence (the reverse complement of the sequence in the U2 snRNA). Figure created with BioRender.com.

The hypothesis that TLR pathway genes that produce negatively acting splice forms are poised to undergo alternative splicing is supported by the analysis of the intron preceding the skipped exon 2 in MyD88-S. Key sites that regulate splicing include the AG at the 3’ end of the intron and the polypyrimidine (pY) tract that lies just upstream of this dinucleotide; the U2AF spliceosome component binds to these sites (129). Upstream of these sites is the branch point, which interacts with the U2 snRNP and associated factors, including the SF3A and SF3B complexes (129). The pY tract in exons that do not undergo exon skipping have relatively strong pY tracts, as evidenced by more thymidine and cytidine residues (130, 131). The pY tract in MyD88 intron 1 has a fair number of thymidine and cytidine residues, suggesting it is of moderate strength (**Figure 6**). The branch point (identified in (132)) in MyD88 intron 1 differs substantially from the expected sequence (the reverse complement of a stretch in the U2 snRNA) (133), suggesting that this branch point sequence is particularly weak. Conversion of these sequences in MyD88 to the canonical sequences in a minigene construct ablated production of MyD88-S (57), also indicating that the splicing regulatory sequences in these TLR pathway genes are poised to enable efficient alternative splicing to produce these negative regulators.

One interpretation of these data is that these splicing regulatory sequences in TLR pathway genes act as the “thermostat” that makes it relatively easy for signaling to induce splice switching and the termination of inflammation. The observation that even moderate inhibition of any of multiple spliceosome components leads to splice switching and the production of negative regulators is consistent with this possibility. A second, not mutually exclusive possibility is that the expression or activity of one or more of these core spliceosome components is regulated by LPS. Which spliceosome component this could be and how its activity might be modulated is unknown.

It is also possible, perhaps even more likely, that other RNA binding proteins such as members of the SRSF or HNRNP families are regulated by LPS and that these family members in turn regulate alternative splicing of these negative regulators. RNAi-mediated Inhibition of SRSF2 increases MyD88-S production (126), although the mechanism of how SRSF2 does so has not been investigated. It also remains to be seen if SRSF2 impacts the LPS-mediated induction of MyD88-S levels.

As described above, much less is known about mechanisms regulating production of other negatively acting isoforms in the TLR signaling pathway. The inclusion of the additional exon between exons 2 and 3 in IRF3a has been examined in one mechanistic study. SR protein family members that are likely to be SRp40 (SRSF5) and SRp55 (SRSF6) bind to an IRF3 pre-mRNA fragment encompassing part of exon 2 and the entire alternate exon 3a (71). Overexpression of these two SR factors increased the ratio of mature IRF3a to IRF3 mRNA confirming their involvement in regulating IRF3 alternative splicing (71). How these factors might be regulated to control IRF3 splicing is unknown. The splicing factors hnRNP A1/A2 and SF2/ASF (SRSF1) regulate the inclusion of IRF3 exons 2 and 3 into the mature mRNA by binding to IRF3 pre-mRNA intron 1. The negatively acting IRF3f form is generated by an exon 2 skipping event, so it possible that these factors also could regulate production of negative IRF3 splice forms, although this has not been tested explicitly (134).

Beyond these few studies examining the mechanisms controlling the alternative splicing of MyD88 and IRF3, little is known about how these negatively acting isoforms are generated. Moreover, a related open question is how much of the mechanism controlling alternative splicing of these negative regulators is shared: is there one unifying mechanism or are there 13 different mechanisms? The answer probably lies somewhere in between, but for now remains a mystery.

## Do these negative regulators affect disease?

The observation that most of these negative regulators are induced by inflammation and thus act as a negative feedback loop suggests that this is a key mechanism that in healthy individuals prevents persistent TLR signaling and chronic inflammatory disease. Moreover, a corollary to this postulate is that the many individuals with infectious or inflammatory diseases may have defects in this alternative splicing mechanism. For example, over-production of the negatively acting splice forms could hinder the inflammatory response and render an individual more susceptible to infection; conversely, under-production of these negative regulators could contribute to inflammatory disorders. Proof-of-principle that altered production of a negative regulator can affect disease is the identification of alternative splice forms of *TNFAIP3*, which encodes the A20 negative regulator. Mis-splicing of this gene leads to loss of production of this negative regulator, which leads to enhanced NF-κB activation and contributes to inflammatory diseases including Still’s Disease and rheumatoid arthritis (135, 136).

What about the effect of negatively acting splice forms in the TLR signaling pathway? As described above, the IKKγΔ/NemoΔ isoform that lacks exon 5 acts in a complex fashion, acting as either a negative regulator or positive regulator of signaling depending on the precise conditions monitored (62–64). This isoform was first described more than 15 years ago (62). Over the years, numerous loss of function mutations have been identified in *NEMO* that present with heritable immunodeficiency (12, 137). Recently, a distinct autoinflammatory syndrome has been identified in which patients harbor mutations that induce NEMO exon 5 skipping; this syndrome has been termed NEMO-NDAS (for NEMO deleted exon 5 autoinflammatory syndrome) (64, 138, 139). NEMO-NDAS in immune cells leads to increased NF-κB activation and type I interferon production. This leads to systemic inflammation, panniculitis, and upregulated interferon regulated genes (64, 138, 139). So again, the effect of this alternate splice form is complicated, and it seems to be more a change of function than negatively acting isoform, at least in many contexts.

Several association studies have been performed to test if alternative splicing in this pathway might affect human disease (**Table 2**). These small association studies suggest that changes in splicing in the TLR signaling pathway could contribute to infectious or inflammatory disease. While human association studies, by their nature, cannot demonstrate causality, they do allow for the assessment of the potential importance of this negatively acting alternative splicing mechanism. These studies also demonstrate the variety of tissues and cell types that produce these negative regulators ranging from T cells in patients with COPD (147) to monocytes in patients with sepsis (145, 146).

**Table 2:** Alternative isoform production associates with the incidence of infectious or inflammatory diseases.

Gene	Pro	Anti	Association	Both?	Reference
MyD88	MyD88-L	MyD88-S	MyD88-L but not MyD88-S upregulated in PBMCs from ARDS patients	Yes	(140)
MyD88	MyD88-L	MyD88-S	MyD88-L but not MyD88-S upregulated in PBMCs from ILD patients undergoing an acute exacerbation	Yes	(140)
MyD88	MyD88-L	MyD88-S	HIV-1 exposed seronegative individuals: MyD88-L:MyD88-S ratio increased following TLR7/8 stimulation of PBMCs	Yes	(141)
MyD88	MyD88-L	MyD88-S	Major depressive disorder: MyD88-S downregulated compared to healthy controls in PBMCs/monocytes	No	(142–144)
MyD88	MyD88-L	MyD88-S	MyD88-S upregulated in monocytes from septic patients	No	(145)
MyD88	MyD88-L	MyD88-S	No change in MyD88-S in monocytes from septic patients with severe melioidosis. No difference in MyD88-S in survivors vs non-survivors	No	(146)
MyD88	MyD88-L	MyD88-S	MyD88-S upregulated in COPD patient CD4^+^ T cells stimulated with αCD3/αC28 & IL12.	No	(147)
MyD88	MyD88-L	MyD88-S	No change in MyD88-L:MyD88-S ratio in human monocytes “tolerized” *in vitro*	Yes	(148)
MyD88	MyD88-L	MyD88-S	MyD88-L increased, MyD88-S unchanged in B cell lymphomas	Yes	(149)
IRAK1	IRAK1	IRAK1c	IRAK1 splicing unchanged in PBMCs from ARDS patients. IRAK1c in PBMCs associated with decreased 28 day mortality in ARDS patients.	Yes	(140)
TLR4	TLR4	Unnamed	Reduced ability to upregulate a possibly negatively acting isoform of TLR4 in LPS-stimulated monocytes from subjects with Cystic Fibrosis	Yes	(87)
TBK1	TBK1	TBK1s	TBK1s but not TBK1 upregulated in PBMCs from HCV-infected patients	Yes	(66)

“Pro” is name of the canonical pro-inflammatory isoform. “Anti” is the name of the alternative splice form that encodes the negative regulator of signaling. Both refers to whether both isoforms were measured or only the anti-inflammatory form was measured in that study.

The alternative splicing of MyD88 and IRAK1 has been examined in a cohort of patients with Acute Respiratory Distress Syndrome (ARDS). The underlying cause of ARDS is some type of lung trauma, often infection (150–155); ARDS patients exhibit significant inflammation in their lungs and systemically (151–153). Even though LPS induces the production of MyD88-S and IRAK1c in cell lines, there were differences in production of these two negative regulators in patients with ARDS. In peripheral blood mononuclear cells (PBMCs) from ARDS patients, MyD88-L but not MyD88-S levels were increased compared to PBMCs from control subjects (140). Perhaps this increase in the MyD88-L to MyD88-S ratio could contribute to the significant inflammation present in these patients. In contrast, IRAK1 splicing was not altered in these patients PBMCs (140). Despite this, it was IRAK1 splicing that correlated with patient outcome. Those patients with more IRAK1c in their PBMCs early after they were admitted to the Intensive Care Unit (ICU) were more likely to survive (140). This suggests that the basal level of the anti-inflammatory IRAK1c isoform could be protective in these patients.

A similar shift in MyD88 splicing was observed in a cohort of patients with a second inflammatory lung disease. MyD88 splicing in PBMCs was also shifted in a pro-inflammatory direction in patients with Interstitial Lung Disease (ILD) who were undergoing an acute exacerbation (140). This condition is associated with significant inflammation and substantial mortality (156). Interestingly, IRAK1 and IRAK1c levels were both significantly increased in this cohort, suggesting that IRAK1 transcription rather than splicing is altered in this inflammatory lung disease.

Several studies have examined the production of MyD88-S in patients with sepsis. Sepsis is a systemic inflammatory disorder that also involves significant immune dysfunction (157–159). MyD88-S was increased in monocytes isolated from septic patients when compared to healthy controls; moreover, this was specific to sepsis, as MyD88-S levels were not increased in a second critically ill cohort, patients who recovered after cardiac arrest (145). Unfortunately, as is true for many studies, MyD88-L levels were not reported in these patients, so it is unclear if this represents a change in splicing or transcription. The authors of this study speculate that the increased MyD88-S levels in these patients could contribute to the immunosuppression present in septic patients. In contrast, MyD88-S levels were unchanged in a different sepsis cohort (one in which sepsis was induced by *B. pseudomallei* infection) (146). Moreover, human monocytes that were tolerized *in vitro* did not exhibit a change in the MyD88-L:MyD88-S ratio (148). So the relationship between MyD88 splicing, sepsis, and immunosuppression remains a complicated one, perhaps not surprising given the extreme complexity of this disease.

MyD88 splicing changes have been observed in other disease cohorts as well. MyD88-L but not MyD88-S levels were increased in B cells from patients with B cell lymphomas (149). The MyD88-L:MyD88-S ratio was increased in PBMCs from individuals exposed to HIV but who remained seronegative, which raised the speculation that a potentially enhanced inflammatory response in these individuals played a role in their ability to avoid disease (141). Likewise, MyD88-S is upregulated in stimulated CD4^+^ T cells from patients with Chronic Obstructive Pulmonary Disease (COPD) (147). Changes in MyD88-S level have also been reported in diseases that are less obviously associated with infection or inflammation. For example, major depressive disorder has been linked to an increase in TLR signaling and inflammation (160, 161), and MyD88-S levels are reported to be decreased in PBMCs and monocytes from these patients (142–144).

While MyD88-S has been the negatively acting isoform most examined in these association studies, there is a little data available about other negative regulators. TBK1s levels but not wild type TBK1 levels were increased in PBMCs from patients infected with Hepatitis C Virus (HCV) (66). A putative negatively acting splice form of TLR4 has been examined in monocytes from a cohort of subjects with Cystic Fibrosis (CF) (87). This isoform of TLR4 is thought to mimic the mouse smTLR4 isoform and produce a truncated soluble decoy receptor, although this has not been tested experimentally. The putative negatively acting isoform of TLR4 is induced upon LPS stimulation in control monocytes but not monocytes from individuals with CF (87). The absolute levels of the putative pro-inflammatory TLR4 isoform were higher in CF monocytes while the putative negatively acting isoform was unchanged (87), suggesting that the basal level of these isoforms differed. Alternative splicing of TLR4 has also been associated with survival in patients with chronic lymphocytic leukemia, although it is unknown what the function of these alternate isoforms is and how they affect leukemia pathogenesis (162).

Numerous cell line studies using overexpression or RNAi indicate that production of these negatively acting isoforms can regulate inflammation. These association studies further suggest the potential importance of this regulatory mechanism in the context of infectious and inflammatory disease. Ultimately, it will be necessary to manipulate splicing of these gene *in vivo* to assess the effect of this alternative splicing in a system where causality can be demonstrated. As yet, few such *in vivo* studies have taken place.

Vickers et al. (82) used antisense oligonucleotides that induce MyD88 exon 2 skipping to increase MyD88-S levels in mice. They repeatedly dosed mice *via* intraperitoneal injection with oligonucleotides that increased MyD88-S production. Following this treatment, they challenged the mice with an intravenous injection of IL-1β. While an intriguing therapeutic modality, they only analyzed one *in vivo* marker of inflammation, and reported that SAA-1 mRNA was decreased in liver of these mice. It would be interesting to examine the extent of the anti-inflammatory effects of modulating MyD88 splicing *in vivo* in this fashion using their described reagents.

A more thorough analysis has been performed assessing the anti-inflammatory effect of MD-2s expression *in vivo* (163). This study used an engineered adenovirus to overexpress MD-2s in mouse lungs (163). Expression of MD-2s *in vivo* significantly weakened LPS-induced lung inflammation as assessed by protein leakage, inflammatory cell recruitment, and production of inflammatory cytokines. Similarly, MD-2s expression also served a protective role in a house dust mite (HDM) sensitization model; MD-2s expression before HDM treatment decreased subsequent markers of allergic airway inflammation. These lung MD-2s studies are proof of principal that manipulating production of these negative regulators could have clinical benefit.

In summary, there are numerous association studies that suggest that production of these negatively acting splice forms could impact infectious or inflammatory diseases. In contrast, very few studies have tested the effect of these negative regulators *in vivo* in mouse experiments designed to test causality. Thus, while these studies are very suggestive, many questions remain about the effect of these negatively acting splice forms in disease pathogenesis.

## Open questions

In many ways, there are more questions than answers about the negatively acting splice forms in the TLR signaling pathway. To start, it is unclear if there are other negatively acting isoforms remaining to be discovered in this pathway. The TLR signaling pathway has been extensively studied because of its functional significance. The reported divergent ratios of some of these isoforms may make it difficult for them to be found in unbiased RNAseq studies depending on the read coverage and the nature of the analysis. Thus, it is perhaps not surprising that most of these inhibitory isoforms have been identified on a gene-by gene basis. Thus, we expect that there very well could be more negatively acting isoforms in this pathway that have not yet been identified. Moreover, while we have focused on the TLR signaling pathway in this review, there are examples of negatively acting splice forms in other innate immune signaling pathways. For example, alternative splice forms of NOD2 and MAVS that encode negative regulators have been identified (164, 165). So this mechanism could be a more generalizable phenomenon because it is so critical that potentially deleterious inflammation be kept in check.

The precise mechanisms used by most of these negative regulators to inhibit signaling are still unclear. In many cases, there are only a few or in some cases even one publication describing these negatively acting splice forms, so there is still lots of mechanistic investigation to be done. Similarly, the mechanisms mediating production of these negative regulators in the first place are largely uninvestigated. How do PAMPs induce production of these negative regulators? Is there a common mechanisms that mediates their production or are multiple mechanisms involved. There is much still to uncover.

From a disease standpoint, the published association studies suggest that defects in production of these negative regulators could be playing a role in human disease. While suggestive, it will be necessary to test these possibilities further by using mouse models that allow for manipulation of these splice forms. While manipulating individual splice forms is more complicated than knocking out a whole gene, there are a variety of mouse genetic tools including CRISPR/Cas9 available for these purposes. If evidence continues to accumulate that these splice forms do play an important role in human disease, then the ability to use splice-switching oligonucleotides to artificially manipulate splicing in this pathway could become an intriguing approach to facilitate mouse disease model studies and potentially a useful therapeutic approach to explore.

Finally, there is an over-arching question of why are there so many negative regulators. This is a question that covers not just these negatively acting splice forms but the myriad of other negatively acting proteins induced following PAMP challenge. Often, ablation of individual negative regulators in mice leads to autoimmune or autoinflammatory phenotpypes. Will the same be true of these negatively acting splice forms or will there be redundancy among them? This becomes particularly relevant as the functional studies on many of these negative regulators were performed using isoform overexpression (although a few studies did use siRNA-mediated inhibition to examine loss-of-function phenotypes). How all these negative regulators function singly and in concert in a physiological setting is a fascinating yet complicated open question.

There is hope that these answers will be forthcoming, as a growing number of studies have been examining alternative pre-mRNA splicing in different contexts including the regulation of innate immunity.

## Author contributions

FL and SA both conceived of, wrote, and edited the manuscript. Both authors contributed to the article and approved the submitted version.

## Funding

This manuscript was supported by NIH grants R01HL148335 and R01AI155749.

## Conflict of interest

The authors declare that the research was conducted in the absence of any commercial or financial relationships that could be construed as a potential conflict of interest.

## Publisher’s note

All claims expressed in this article are solely those of the authors and do not necessarily represent those of their affiliated organizations, or those of the publisher, the editors and the reviewers. Any product that may be evaluated in this article, or claim that may be made by its manufacturer, is not guaranteed or endorsed by the publisher.
